# Simple and Efficient 3D-Printed Superfusion Chamber for Electrophysiological and Neuroimaging Recordings *In Vivo*

**DOI:** 10.1523/ENEURO.0305-22.2022

**Published:** 2022-10-04

**Authors:** Dmitrii Suchkov, Viktoria Shumkova, Violetta Sitdikova, Marat Minlebaev

**Affiliations:** 1 Institut de Neurobiologie de la Méditerranée, Institut National de la Santé et de la Recherche Médicale, Aix-Marseille University, Marseille, France; 2 Laboratory of Neurobiology, Kazan Federal University, Kazan, Russia

**Keywords:** 3D printing, drugs screening, electrophysiology, neuroimaging, *in vivo*

## Abstract

*In vitro* and *in vivo* experimentation in the central nervous system are effective approaches to study its functioning. Manipulations *in vitro* are characterized by easy experimental control and stable experimental conditions. However, transferring these advantages to *in vivo* research remains technically and ethically challenging, preventing many research teams from acquiring critical recordings in their animal models. In order to transfer the benefits of *in vitro* experimentation to *in vivo* experimentation, we developed a suite of 3D-printed tools (a superfusion chamber with an independent brain presser and animal stand). Using the immature rat barrel cortex as a model, we show that our set of tools (further “superfusion preparation”) provides stable conditions for electrophysiological and neuroimaging recordings in the neonatal rat neocortex *in vivo*. Highly correlated intracellular and extracellular activity was recorded during spontaneous and evoked cortical activity, supporting the possibility of simultaneous long-lasting electrophysiological recordings from a single cortical column *in vivo*. The optical intrinsic signal of evoked cortical responses was also recorded from the skull-free neocortex, suggesting the effective combination of the superfusion preparation with neuroimaging approaches. Modulation of immature activity by epicortical application of pharmacological agents via superfusion equally supports the use of the superfused cortex preparation in pharmacological screening. In addition to high efficiency (in affordability, reliability, and ease of use *in vivo*), the 3D-printed set of tools developed should reduce animal use, supporting the 3Rs principle (Replacement, Reduction, and Refinement) of ethical use of animals.

## Visual Abstract

**Figure:**
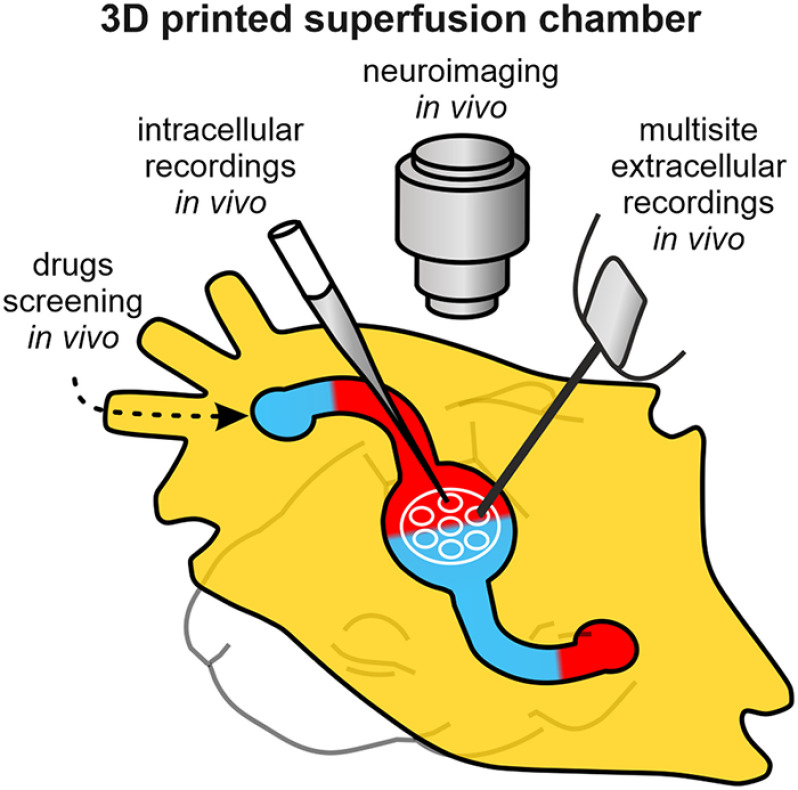

## Significance Statement

This study describes a 3D-printed superfused chamber developed for extracellular/intracellular and neuroimaging cortical recordings *in vivo*. The device consists of a set of independent parts (a superfusion chamber with an independent brain presser and animal stand) that serve for stable, unified, and well-controlled experimental manipulations in the living rat. In addition to facilitating experimental procedures, the proposed superfusion chamber allows drug screening that also supports the 3Rs principle (Replacement, Reduction, and Refinement).

## Introduction

Despite the development of methods that can substitute experimentation on living neural tissue, such tissue is still required for studying the function of the central nervous system. Both *in vitro* and *in vivo* experimentation are efficient techniques in neuroscience to study the brain. Although *in vivo* studies are the most realistic model for understanding the functioning of the living brain, *in vitro* experimentation is a proven method characterized by high availability and tight control of the experimental conditions. To transfer these advantages to *in vivo* studies, a great number of devices and approaches have been designed. But there has been little work toward the improvement of manipulation of the animal.

Electrophysiological recording is the gold standard in the study of local and brain-wide microcircuit electrical activity. Although electrical neuronal activity reaches hundreds of uV in amplitude, single neurons and restricted neuronal networks generate small and fast fading electric potentials (Mitrukhina et al., 2015). Such tiny signal recordings require stable positioning of the electrode close to the active neuronal cells. Although the electrode is fixed in space, respiration, heartbeats, and animal movement produce brain pulsations, impairing the quality of the recording.

To fix the pulsation problem, the size of the open cortical area is limited to leave only enough space to insert the electrode (Khazipov et al., 2004; Hanganu et al., 2006; Minlebaev et al., 2007; Yang et al., 2009). While this solution perfectly solves the pulsation problem for extracellular recordings, it is a key limitation for intracellular recordings, for which there is a relatively low success rate to patch a cell with a single insertion of a glass electrode.

There have been attempts to preserve brain immobility and to enlarge the accessible cortical area, by replacing the part of the skull with an artificial chamber over the region of interest (Khazipov and Holmes, 2003; Minlebaev et al., 2007; Tyzio et al., 2008).

The skull opening also serves to give visual access to the cortex to improve neuroimaging recordings. To prevent brain pulsation, the opened surface is covered with a highly transparent material (Ferezou et al., 2007; Yang et al., 2013; Heo et al., 2016).

Testing the action of pharmacological agents has had a great impact in the study of the mechanisms of physiological brain functioning, and in the development of methods of treatment of pathologic brain conditions. A particularly important approach is direct drug application to the brain to avoid bias by drug systemic effects and secondary side effects (Khazipov and Holmes, 2003; Minlebaev et al., 2007, 2009). However, despite attempts to improve brain recordings *in vivo*, there is still complexity to overcome to transfer the advantages of *in vitro* recordings to *in vivo* studies.

Here, we present a device that simplifies electrophysiological and neuroimaging recordings *in vivo*. The device is made by 3D-printing technology using commercially available 3D fused deposition modeling (FDM) and digital light processing (DLP) printers. It is low cost, small, and gives high reliability and precision between experiments. We show that the proposed device is a reliable solution for stable, unified, and well-controlled *in vivo* recordings.

## Materials and Methods

### Ethical approval

All animal-use protocols followed the guidelines of Kazan Federal University on the use of laboratory animals (ethical approval by the Institutional Animal Care and Use Committee of Kazan State Medical University N9-2013).

### Superfusion chamber setup

Most parts of the superfusion chamber were printed on Ender-3 Pro (Creality 3D) and PRUSA I3 MK3S+ (Prusa Research) 3D printers using FDM technology. The free online 3D editor Tinkercad (Autodesk) was used to design the parts. Links to the 3D models can be found in Table 1. These are ready-to-print component files that can be imported into any 3D-printing slicer. M4x35 screws and M4 nuts were used to mount the chamber on the stand. Flex silicon tubes (Tygon) were used to connect the parts of the perfusion system. The approximate cost of the parts is shown in Table 2. The chamber has a small volume of 250 μl for better control of the temperature of the superfusion solution. The upper diameter of the chamber is bigger than the bottom diameter (16 and 8 mm, respectively) to provide easier access from the side. The chamber has inner channels to maximize the accessible area of the chamber. These channels have a curved shape that reduces the fluctuations of the surface of the liquid, allowing stable OIS recordings. To minimize the number of parts, the chamber has connectors with outer and inner diameters of 2.6 and 1.5 mm, respectively, to be connected directly with the perfusion tubes. Two thermosensors were also incorporated into the chamber (at the input and output) to measure the temperature of the superfusion solution, that is, heated before the chamber. Stabilization and regulation of the solution level were realized via a movable separate suction part. The suction part connected to the output of the chamber via a Tygon tube independently fixed with a one-directional *z*-axis manipulator. Moving the suction part down resulted in reduction of the solution volume in the chamber.

**Table 1:** Summary of all design files for the superfusion chamber

Design file name	File type	Open-source license	Location of the file
Superfusion chamber	STL	GNU GPL v3	Superfusion chamber
Connector	STL	GNU GPL v3	Connector
Regulation chamber	STL	GNU GPL v3	Regulation chamber
Stereotaxis plate	STL	GNU GPL v3	Stereotax is plate
Support column	STL	GNU GPL v3	Support column
Presser	STL	GNU GPL v3	Presser
Presser holder	STL	GNU GPL v3	Presser holder

**Table 2:** Complete bill of materials for producing the superfusion chamber setup

Component	Number	Cost per unit	Material type
Superfusion chamber	1	$0.06*	PLA
Out connector	2	$0.01*	PLA
Regulation chamber	1	$0.01*	PLA
Stereo taxis plate	1	$0.62*	PLA
Support column	3	$0.02*	PLA
Presser	1	$0.01*	PLA
Presser holder	1	$0.02*	PLA

* the price is approximate.

### Surgery

Male and female Wistar rats from postnatal day (P)4 to P7 (21 rat pups), and three rats P20–P27 were used. The surgery was performed under isoflurane anesthesia (5% for the induction and 1.5% during the surgery). After an incision to remove the scalp, the skull was cleaned and covered with cyanoacrylate adhesive glue except for a 50-mm^2^ window above the cortex for electrode placement. Using dental cement, the superfusion chamber was fixed to the skull centered on the barrel cortex. In the experiments with an opened cortical surface, a 5-mm-diameter burr hole was drilled in the skull above the barrel cortex, the dura was gently cut and removed, and the cortical surface was covered with 0.9% NaCl to prevent it from drying. After the surgery the animal was surrounded by a cotton nest and heated via a thermal pad (35–37°C) and left to recover from anesthesia for 1 h. Afterward, a chloride-coated silver wire was placed in the cerebellum or visual cortex and served as the ground electrode. All recordings were made from rats anesthetized by intraperitoneal injection of urethane (0.5–1.5 g/kg). Urethane was chosen because of its weaker modulation of physiological patterns of activity (Shumkova et al., 2021). The cortical surface was superfused with oxygenated (95% O_2_-5% CO_2_) artificial cerebrospinal fluid (ACSF) of the following composition: 126 mm NaCl, 3.5 mm KCl, 1.2 mm NaH_2_PO_4_, 2 mm CaCl_2_, and 1.3 mm MgCl_2_, throughout the whole experiment. The temperature in the chamber was kept at 35–37°C using an automatic temperature controller (TC-344B; Warner Instruments).

### Stimulation

Before the experiment, whiskers were trimmed to a length of 3 mm. The principal whisker (PW) was stimulated by piezo deflection. The tip of the whisker was inserted 2 mm into a wire ring attached to the piezo bender (Noliac) so that the whisker rested snugly inside. To induce deflection of the piezo bender, square 50- to 70-V pulses of 10 ms were applied. To avoid depression of the evoked response, whiskers were stimulated every 50 s.

### Optical intrinsic signal imaging

Optical imaging of Intrinsic Signal (OIS) was performed using the following video acquisition system. The camera (QICAM Fast 1394, Qimaging) was positioned orthogonally to the exposed skull above the estimated location of the somatosensory cortex. To detect the OIS, the camera was focused using a system of objectives at 0.4–1.2 mm (depending on animal age) below the skull to the expected depth of the thalamorecipient layer of the barrel cortex. To estimate changes in blood flow green/red (OSRAM, LRT GFTM-ST7-1+VV9-29–0-A-R33-ZB, 520/610 nm) highlighting diodes were placed above the animal’s head. The reflected light was collected by a CCD camera (QICAM Fast 1394, 130 × 174 resolution, 1 pixel = 35 μm). Because of the low amplitude, the OIS was calculated based on the average of 20 repeated video acquisitions. Each repetition had a duration of 100 s with a 5-s prestim period followed by a 2-Hz train of whisker deflection (10-ms duration each) for 10 s with 90 s of recovery. During preprocessing, the recorded video was downsampled to 10 Hz, followed by OIS detection in which an averaged intrastimulus video frame was compared with an averaged prestimulus video frame. OIS detection was performed manually by the operator by masking off the area with decreased light intensity. All values from the reference region that exceeded the significance level of 2.5 Jackknife SD of noise were considered as the OIS (for the details of OIS denoising and detection, see Sintsov et al., 2017). Only results for the green diode were used to demonstrate the applicability of the OIS technique.

### Extracellular recordings

The coordinates of the cortical whisker representations of interest were determined using OIS imaging as described above. Extracellular recordings of evoked and spontaneous cortical activity in the cortical whisker representations of interest were performed using a single shank 16-channel silicon probe (Neuronexus Technologies) with a separation distance of 0.1 mm between electrodes. The recording electrode was aligned perpendicular to the skull surface to reach the barrels of interest. The upper electrodes were placed at the depth of the granular layer of the sensory-recipient barrel which was confirmed by the combination of the following factors: (1) presence of a short latency evoked local field potential (LFP) deflection and multiple-unit activity following stimulation of the corresponding whisker (Minlebaev et al., 2009; Yang et al., 2013; Mitrukhina et al., 2015) and (2) predominance of the γ frequency in the evoked LFP and multiunit activity (MUA; Minlebaev et al., 2011). The lower electrodes were placed at the depth of the infragranular layer. The signals were amplified (×10,000), filtered (0.1 Hz to 10 kHz) using a 128-channel amplifier (Neuralynx) or 64-channel amplifier (DIPSI, France) and analyzed *post hoc*.

### Intracellular recordings

Patch-clamp recordings were performed using an Axopatch 200A amplifier (Molecular Devices) using a patching technique similar to that described in (Leinekugel et al., 2002; Margrie et al., 2002). The pipettes were filled with a solution of the following composition: 135 mm Cs-gluconate (or methylsulfate), 2 mm MgCl_2_, 0.1 mm CaCl_2_, 1 mm EGTA, and 10 mm HEPES (pH 7.25). Membrane potential values were corrected for liquid junction potential of +12 mV. Data were digitized at 10 kHz using a Digidata 1440A interface (Molecular Devices) and analyzed offline using self-made functions in MATLAB (MathWorks).

### Pharmacological application

The agents 6-cyano-7-nitroquinoxaline-2,3-dione (CNQX, Sigma) and d-2-amino-5-phosphonovaleric acid (D-APV, Sigma) were diluted in ACSF (40 μm CNQX and 160 μm D-APV) and applied to block glutamatergic transmission. The application was made as a single load of 250 μl of solution with superfusion stopped. Within 25 min, visual observation showed suppression of the cortical activity, that was recorded for further analysis. Then superfusion was switched on to wash out CNQX and D-APV. In 20–30 min, the brain activity recovered and was recorded. This was followed by a pause in superfusion to load 250 μl of bicuculline (100 μm) to block GABAergic transmission. Although the first interictal activity was observed several minutes after bicuculline application, the recordings were started after 10–20 min.

### Data analysis

Raw data were preprocessed using custom-written functions in MATLAB (MathWorks). Raw data were explored to detect MUA, followed by raw data downsampling to 1000 Hz for further analysis of LFP. The analysis was conducted in a 500-ms windows after the stimulus to define evoked activity (Suchkov et al., 2016). MUA was detected in a band-passed signal (>200 and <4000 Hz) in which all negative events exceeding 3.5 SDs calculated over the entire trace were considered as spikes (>99.9% confidence; Mitrukhina et al., 2015). Activity following the stimulation with onset delay of <40 ms was considered as evoked (Minlebaev et al., 2011), episodes of oscillatory activity observed with a delay of 1 s after the stimulus were analyzed as spontaneous activity. Spontaneous activity in neonatal rat pups was detected using analysis of MUA density. Analysis consisted of two stages. First, the logarithmic distribution of the intervals between MUA was approximated by the combination of two Gaussian distributions. That was done assuming the presence of short episodes of cortical activity separated by long silent intervals. Each Gaussian distribution represents short-time and long-time intervals between MUA, respectively. MUA with intervals shorter than the mean value for the short-time distribution were combined into MUA groups. Onset times for the MUA groups was used as new pseudo-MUA activity. The procedure described above, was repeated for the pseudo-MUA activity until the distance between pseudo-MUA was less than the predefined value of 100 ms. Thus, the final composition of groups was defined as a set of activity episodes. Additionally, activity episodes were clustered using the number of MUA in each episode, duration of the episodes, and median MUA interdistance inside each episode. Only episodes with large values were used. Finally, the episodes detected were verified by the operator to exclude artifacts.

The spectral parameters for MUA were evaluated using Chronux toolbox (Bokil et al., 2010). Spectral power of the LFP was calculated using wavelet transform. As a signal’s spectral power at each frequency is largely related to that frequency by a power law, the ratio between spectral powers of the target signal and baseline was used to characterize the power spectral density of the analyzed signal.

Cross-correlation was done referring to the LFP troughs and calculated using the peak times of the evoked postsynaptic currents recorded during the 0.5-s window following the stimulus.

OIS onset/offsets were calculated based on the crossings of the linear approximation of the rising/falling OIS phases and zero amplitude level. Duration of the OIS was defined as the difference between offset and onset.

All distributions of the parameter values were checked for normality using the Lilliefors test. For normal distributions, a confidence interval was evaluated and based on the *t* Student’s criteria. A box plot of statistics was presented for non-normal distributions.

The nonparametric Wilcoxon rank sum test was used to estimate if samples are from continuous distributions with equal medians; *p*-values higher than 0.05 represent a nonsignificant difference and marked as ‘ns’ on figures.

## Results

### Recordings of electrical activity using the superfusion chamber *in vivo*

#### Extracellular recordings of cortical activity

Electrical activity was recorded from a single cortical column that was detected using the OIS technique (Sintsov et al., 2017).

**Figure 1.**
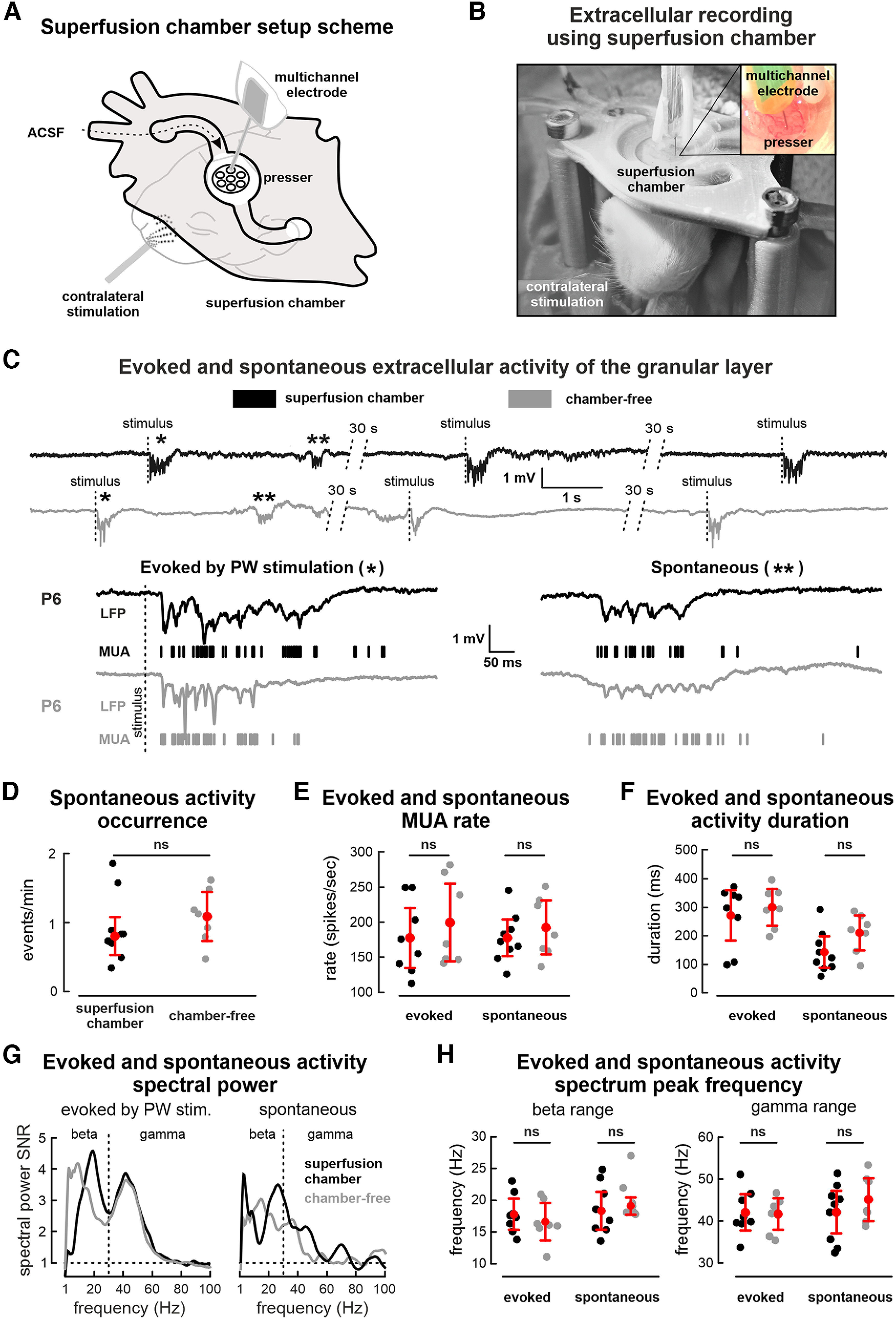
Electrophysiological extracellular recordings using the superfused cortex preparation. ***A***, ***B***, Scheme and picture of the superfused cortex preparation with a presser fixed on the rodent head. ***C***, Evoked and spontaneous neuronal network activity recorded in the barrel cortex with (black trace) and without (gray trace) the superfusion chamber. Expanded episodes of evoked (left) and spontaneous (right) activity marked by a single and doubled asterisk on the traces are shown below. Lines correspond to the fluctuations of LFP, while vertical bars are MUA. ***D***, Group data of spontaneous activity occurrence recorded with and without the superfusion chamber. ***E***, ***F***, Group data of MUA rate during episodes of evoked activity and its duration recorded with and without the superfusion chamber. ***G***, Power spectral density of evoked activity recorded with and without the superfusion chamber. ***H***, Group data of evoked activity spectral peak distribution in the β and γ frequency ranges recorded with and without the superfusion chamber. Filled circles represent the results of individual experiments; red circles, mean values; red whiskers, confidence intervals based on *t* Student’s criteria.

Using the superfusion chamber both evoked and spontaneous activity were recorded for further analysis (Fig. 1*A*–*C*). The short-lasting single whisker deflection on the whisker snout of the animal evoked an oscillatory response lasting up to several hundreds of milliseconds. A comparison of the evoked and spontaneous activity recorded with the superfusion chamber and without it did not show any significant difference in LFP and MUA characteristics (Fig. 1*D–H*). The occurrence of spontaneous oscillatory events weakly differed between conditions (with or without superfusion chamber, 0.8 ± 0.3 events/min, *n* = 9, P5–P8 rats and 1.1 ± 0.4 events/min, *n* = 7, P4–P7 rats, *p* = 0.15, respectively; Fig. 1*D*). Analysis of the neuronal firing associated with the evoked response (182 ± 44 units/s, *n* = 8 P5–P8 rats) or spontaneous events (183 ± 27 units/s, *n* = 9 P5–P8 rats) also did not significantly differ from that recorded in chamber free animals (206 ± 56 units/s and 198 ± 40 units/s, respectively, *p* = 0.46, *n* = 7 P4–P7 rats; Fig. 1*E*). The duration of the evoked responses recorded with the superfusion chamber (338 ± 112 ms, *n* = 8 P5–P8 rats) was not significantly different (*p* = 0.78) from that in chamber free animals (374 ± 81 ms, *n* = 7 P4–P7 rats). There was also no significant difference (*p* = 0.07) in the duration of the spontaneous responses between the superfusion chamber (175 ± 70 ms, *n* = 9 P5–P8 rats) and the standard preparation (260 ± 77 ms, *n* = 7 P4–P7 rats; Fig. 1*F*). Analysis of power spectral density for spontaneous and evoked activity showed spectral peaks in the β (14–30 Hz) and γ (30–100 Hz) frequency ranges (Fig. 1*G*). The peaks for evoked and spontaneous activity were at 18 ± 2 and 19 ± 3 Hz for the β and 42 ± 4 and 42 ± 5 Hz for the γ frequency ranges in animals with the superfusion chamber, respectively (*n* = 9, P4–P7 rats). These results were not significantly different from the spectral features of the evoked and spontaneous activity recorded in chamber-free animals (17 ± 3 and 19 ± 1 Hz for the β range and 42 ± 4 and 45 ± 6 Hz for the γ range, respectively, *n* = 7 P4–P7 rats; Fig. 1*H*).

Therefore, installation of the superfusion chamber *in vivo* does not modify the physiological patterns of activity in the somatosensory cortex of the neonatal rat.

#### Intracellular recordings

**Figure 2.**
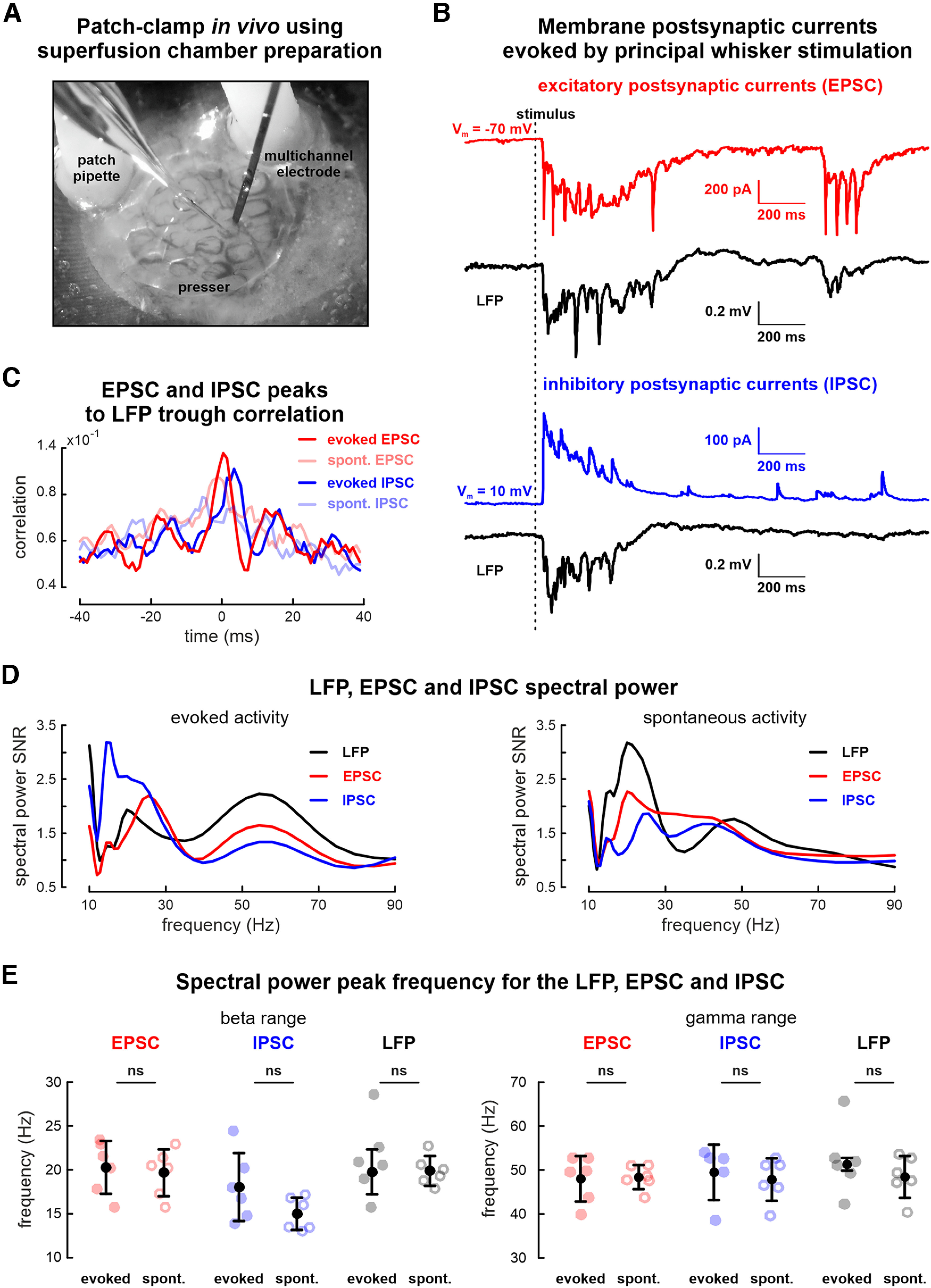
Electrophysiological intracellular and extracellular recordings using the superfusion chamber. ***A***, Picture of simultaneous intracellular and extracellular recording using superfusion chamber. ***B***, Simultaneously recorded evoked EPSCs (red trace), IPSCs (blue trace), and corresponding extracellular LFP responses (black trace) recorded in one cortical column of the barrel cortex. Note that spontaneous activity is also observed both extracellular and intracellularly. ***C***, Cross-correlation between extracellular and intracellular activity recorded using the superfusion chamber. Cross-correlation centered on LFP troughs. ***D***, LFP (black line), EPSC (red line), and IPSC (blue line) spectrum of the evoked response during PW stimulation. ***E***, Group data for the spectrum peak frequencies in the β (left) and γ (right) frequency range. Peak frequency values of the evoked and spontaneous response for the EPSC (light red filled and empty circles, respectively), IPSC (light blue filled and empty circles, respectively), and LFP (light gray filled and empty circles, respectively) are shown. Black filled circles represent mean values; black whiskers, confidence intervals based on the *t* Student’s criteria.

The multisite silicon probe was installed into the column detected by OIS, followed by the cell patching procedure in this area (Fig. 2*A*). Intracellular recordings in voltage clamp configuration showed both spontaneous and evoked EPSCs and IPSCs (Fig. 2*B*). To record IPSCs and EPSCs, the membrane potential was maintained close to the reversal potential of the GABAa-mediated postsynaptic (around −70 mV) and glutamatergic excitatory currents (around 10 mV), respectively. IPSCs and EPSCs were in synchrony with the neuronal network activity recorded extracellularly (Fig. 2*C*).

Neither peak frequencies in the γ frequency range for the evoked and spontaneous EPSCs (47 ± 5 and 47 ± 3 Hz), IPSCs (48 ± 6 and 44 ± 5 Hz), nor simultaneously recorded LFP (50 ± 1 and 47 ± 5 Hz) showed a significant difference in frequency (*n* = 6 cells, *p* = 0.73, *p* = 0.40, and *p* = 0.51, respectively, P5–P7 rats; Fig. 2*E*), supporting the idea that proposed preparation allowed recordings from the same cortical column. There was neither a significant difference between peak frequencies in the β range for the evoked and spontaneous EPSC (20 ± 3 and 20 ± 3 Hz), IPSC (18 ± 4 and 15 ± 2 Hz), nor for simultaneously recorded LFP (20 ± 3 and 20 ± 2 Hz; *n* = 6 cells, *p* = 0.67, *p* = 0.13, and *p* = 0.93, respectively, P5–P7 rats). The patched cells were recorded for up to 1.5 h (1.1 ± 0.4 h, *n* = 6) showing efficient brain immobilization by the independent presser.

Thus, the superfusion chamber with the independent presser allows stable intracellular and extracellular recordings in spatially restricted cortical spots.

### Recordings of the optical intrinsic signal in the superfused cortex preparation

**Figure 3.**
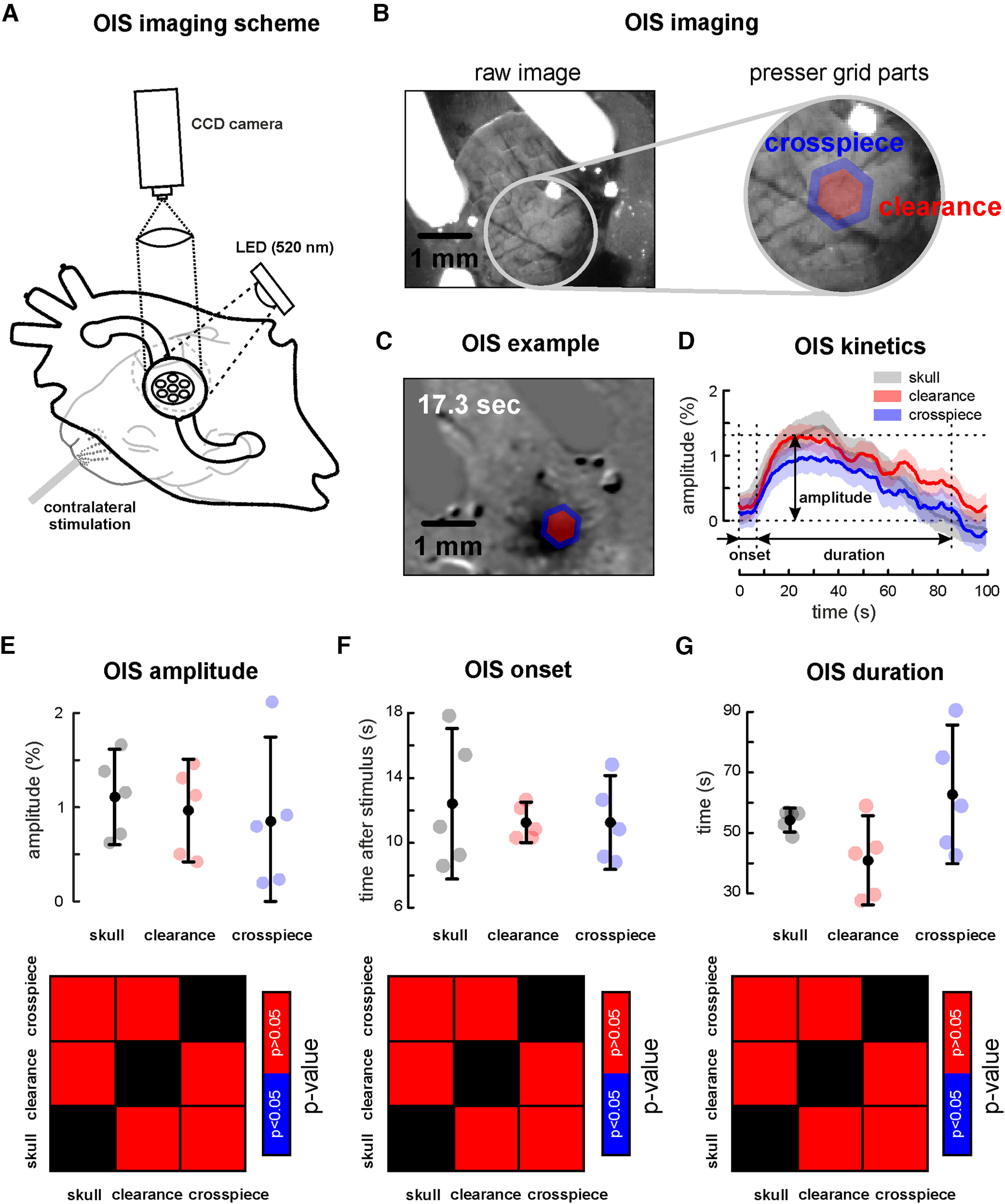
Optical intrinsic signal recordings using the superfusion chamber. ***A***, Experimental scheme of OIS recordings in vivo. ***B***, Field of view of the cortical surface fixed under the presser recorded using the green illumination shown on the left. Expanded view of the presser with clearance (red) and crosspiece (blue). ***C***, OIS evoked by whisker stimulation from the experiment shown in ***B***. Note the crosspiece and clearance zones are over the barrel cortex. ***D***, Comparison of the dynamics of the OISs recorded under the clearance (red), crosspiece (blue), and that recorded through the skull (gray). The shaded area corresponds to the SD of the OIS. ***E***, Group data for the OIS amplitude under the clearance (red), crosspiece (blue), and recorded through the skull (gray). ***F***, Group data for OIS onset in the zone of clearance (red), crosspiece (blue), and through the skull (gray). ***G***, Group data for OIS duration under the clearance (red), crosspiece (blue), and through the skull (gray). Black filled circles represent mean values, black whiskers, confidence intervals based on the *t* Student’s criteria.

Although the OIS method is effective, its low amplitude rarely exceeds 2% of light drop (especially in the developing cortex) and critically depends on the experimental conditions (brain stability, light transparency above the region of interest); therefore, OIS is a perfect tool to test whether our chamber can provide appropriate conditions for neuroimaging recordings. For this purpose, the OIS was recorded twice, before (through the skull) and after chamber installation (with a presser covering the opened cortical area; Fig. 3*A*). The OIS was evoked by whisker stimulation as described previously (Suchkov et al., 2018; Shumkova et al., 2021). As the presser was used, OIS parameters were characterized both under the presser crosspiece and in the clearances between them (Fig. 3*B*,*C*). The OIS was compared in amplitude and time parameters (Fig. 3*D*). The amplitude of the recorded OIS (Fig. 3*C*) was weakly different with and without the chamber (Fig. 3*D*). Conventional OIS recording through the skull was not different both under the crosspiece and in the clearances between them (*n* = 5, *p* = 0.55 and *p* = 0.55, respectively, P5–P8; Fig. 3*E*). That was also true for the comparison of the time features of the evoked OIS signals. Neither evoked OIS onset nor its duration were different in chamber free and superfused cortex preparation conditions of OIS recordings (*n* = 5, *p* = 0.69 and *p* = 0.69 for the crosspiece, *p* = 0.95 and *p* = 0.15 for the clearances, respectively, P5–P8 rats; Fig. 3*F*,*G*; Table 3).

**Table 3:** OIS parameters

OIS parameter	Skull	Crosspiece	Clearance
Amplitude	1.1 ± 0.5%	0.9 ± 0.8%	1.0 ± 0.5%
Onset	12 ± 5 s	11 ± 3 s	11 ± 1 s
Duration	54 ± 4 s	63 ± 23 s	41 ± 15 s

Therefore, the OIS could be effectively recorded using the superfusion chamber with the presser *in vivo*, supporting the idea of superfusion chamber use for neuroimaging *in vivo*.

### Pharmacological manipulations of cortical neuronal activity with the superfused cortex preparation *in vivo*

**Figure 4.**
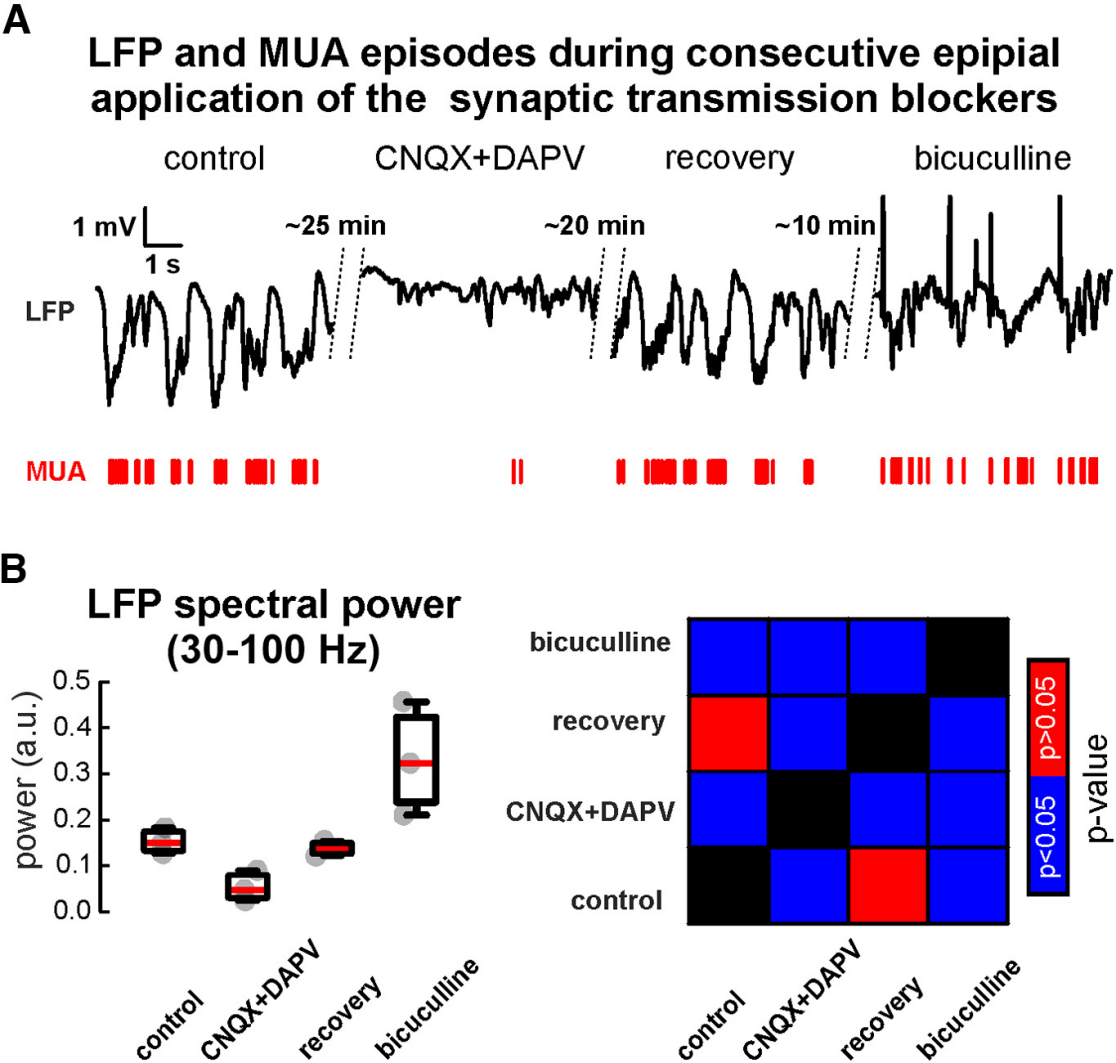
Epipial pharmacological application using the superfusion chamber. ***A***, Cortical granular layer activity recorded in P27 animal in control condition followed by consecutive application of glutamatergic blockers, wash-out and then application of an inhibitor of GABAergic transmission. LFP (black lines) and corresponding MUA (red bars) are shown for each pharmacological condition. ***B***, Group data for the LFP spectral power in γ frequency range (30–100 Hz; calculated using wavelet decomposition) for each pharmacological condition (left panel). Gray-filled circles are the results of individual experiments. On each box, the central mark indicates the median, and the bottom and top edges of the box indicate the 25th and 75th percentiles, respectively. The whiskers extend to the most extreme data points not considered outliers. Nonparameteric Wilcoxon test *p*-values checkboard (right panel) between LFP response spectral power during various pharmacological conditions. Blue color (significant difference) corresponds to *p* < 0.05, while red color (nonsignificant difference) to *p* > 0.05.

We have tested whether it is possible to use the superfusion chamber for the application of pharmacological agents on the surface of the neocortex *in vivo*. Consecutive epicortical application of glutamatergic and GABAergic blockers were used (Fig. 4*A*). Although the application of CNQX (40 μm) and D-APV (160 μm) strongly reduced cortical activity compared with control conditions (*n* = 3, P20–P27; see Table 4), washing the cortical surface with ACSF restored it to the control values. Application of 40uM bicuculline increased the power of the cortical activity *in vivo* (Fig. 4*B*; Table 4).

**Table 4:** LFP power in γ frequency range, a.u.

Condition	Median	25%	75%
Control	0.14	0.13	0.17
CNQX+DAPV	0.05	0.03	0.09
Recovery	0.14	0.13	0.15
Bicuculline	0.32	0.24	0.42

Altogether, our results clearly demonstrate that multiple modulations of cortical activity can be conducted by consecutive applications of different pharmacological actions when using the superfusion preparation.

## Discussion

Here, we demonstrate a do-it-yourself (DIY) combination of devices designed for high turnover brain experiments, printed using commercially available low-cost 3D printers. It is a modular design that consists of a single-use chamber for cortical superfusion and restraining the animal’s head and a multi-use presser and animal stand. Machine-controlled part fabrication much improves reliability and reproduciability between experiments. Standardized parts significantly reduce the manual work needed for experimentation and the animal preparation time.

The two 3D-printing technologies FDM and DLP were used. While both technologies are efficient for chamber manufacture, we highly recommend FDM using PLA filament. The chamber has internal millimeter-diameter tunnels used for fluid flow into and out of the chamber, however even low-cost FDM printers are able to print these tunnels. The two FDM printers used in our laboratory (Ender-3 Pro and PRUSA I3 MK3S+) successfully printed the chambers. Furthermore, PLA is a thermoplastic biodegradable polyester, therefore, PLA-made chambers discarded after a single use have a reduced environmental impact.

For experimental manipulation without the brain presser, only a FDM printer with PLA filament is required. For a single chamber, the approximate weight is ∼2 g, so a 1-kg spool of PLA is enough to print 500 chambers. The animal stand was also printed using FDM. The animal was fixed to the stand via support columns 2 cm in height to keep the neonatal rat pup’s head raised a bit higher than body level (for adult animals columns are up to 3–4 cm).

The chamber and the animal stand provide electrical insulation of the animal from the setup, as PLA has limited conductance.

Compared with FDM, DLP technology has higher spacial precision. We have Anycubic Photon Series printers (Photon S and Photon Mono) with a spatial resolution of 0.05 mm in *xy* space and 0.01 mm in the *z*-axis. Because of the higher spatial resolution, the presser with the tiny mesh was manufactured using DLP technology. The hexagonal organization of the mesh (0.2- to 0.3-μm hexagon side and crosspieces of 0.2-μm thickness) served to minimize the distance between the crosspieces while maximizing the size of the open area. Although the presser has a tiny mesh structure, careful use allows for multiple uses. Therefore, the use of a DLP printer was only justified by the presser, for suppressing brain pulsations in experiments with a large open cortical area.

Free 3D CAD software (Tinkercad) was used to design the devices, allowing further modifications for various experimental needs, such as targeting different brain regions and use in other animal species. We demonstrate the efficiency of the 3D-printed superfusion chamber in electrophysiological recordings *in vivo* using both extracellular and intracellular registration. Previously, the concept of superfused brain preparations was already shown (Khazipov and Holmes, 2003; Minlebaev et al., 2007, 2009; Tyzio et al., 2008). A plastic cylinder made from the top of a syringe or plastic pipette with a stretched mesh glued to it served as a chamber. The chamber was then fixed to the skull with dental acrylic.

Therefore, the successful combination of stable electrophysiological recordings and pharmacological manipulation of the network activity is promising in the study of the functioning of the central nervous system.

But the limited access area and absence of pressure control of the chamber on the brain tissue restrict the use of the chamber in electrophysiological and/or neuroimaging recordings. The combination of a low-profile chamber with an 8-mm diameter open area and the independent presser solves these problems. Moreover, fixing the presser to an independent manipulator allows easier placement and fine control of the force of the presser. We showed that these modifications allow stable simultaneous electrophysiological recording (extracellular and intracellular) of physiological activity while maintaining the previously reported advantages. Moreover, the clearances of the hexagonal mesh allow both extracellular and intracellular recordings from a single cortical column (∼0.2-mm diameter in the barrel cortex). The clearances allow multiple patch trials, which also reduces the number of experiments and animals used to accumulate statistically significant numbers of recordings.

Electrophysiological approaches are the gold standard for cortical activity recordings because of their high temporal resolution. But neuroimaging has higher spatial resolution, making neuroimaging techniques popular and widely spread (Malonek and Grinvald, 1996; Ferezou et al., 2007; Yang et al., 2013; Ackman et al., 2014; Burbridge et al., 2014; Bolaños et al., 2018).

We show that a plastic hexagonal mesh suppresses brain movement and provides the conditions for stable recording of an OIS with similar parameters to an OIS recorded through the skull. Although we only tested a single neuroimaging technique, we hope that the superfused brain preparation is also compatible with other neuroimaging techniques.

Finally, one of the principal advantages of *in vitro* studies is the ability to modulate neuronal activity in a predictable manner. In addition to the previously mentioned features, the superfusion chamber transfers this advantage to *in vivo* conditions. We reported that consecutive application of pharmacological agents with wash-out of the previous drug suggests the high potential of the superfusion chamber for screening studies *in vivo*. Minimizing the number of animals used per experiment or study serves the principle of 3Rs (Replacement, Reduction, and Refinement).

To summarize, the 3D-printed chamber presented here is a low-cost, easily manufactured, highly reliable, and accessible device to increase the efficiency and reproducibility of *in vivo* experimentation.

Its low weight makes the superfusion chamber ideal for acute recordings in rodents. We have verified the chamber in *in vivo* experiments with extracellular and intracellular electrophysiological and wide-field neuroimaging recordings, showing that it is suited for the proposed application. We believe that the accessibility of 3D-printing technology will enable our design to be used and adapted to a variety of experimental procedures.

## Synthesis

Reviewing Editor: Alexey Semyanov, Shemyakin-Ovchinnikov Institute of Bioorganic Chemistry Decisions are customarily a result of the Reviewing Editor and the peer reviewers coming together and discussing their recommendations until a consensus is reached. When revisions are invited, a fact-based synthesis statement explaining their decision and outlining what is needed to prepare a revision will be listed below. The following reviewer(s) agreed to reveal their identity: Yuriy Pankratov.

Your paper has been seen by two reviewers and both have been positive about your work. However, they made several minor comments which need to be addressed before the paper can be suitable for publication.

Reviewer #1

The manuscript describes an innovative low-cost system for in vivo imaging and electrophysiological recordings, including whole cell recordings of synaptic currents. Authors provide substantial evidence of suitability of this system for high-quality and, in perspective, high-throughput neurophysiological experiments in rodents. The manuscript also provides sufficient details and description which would enable any research with average “hands-on” lab skills and experience to replicate this system. Usage of such system would greatly greatly promote in vivo research in physiological mechanisms of synaptic signalling.

In general, the visual abstract is adequate but its presentation quality might be improved further, for instance:

1. The intracellular and extracellular electrodes can be shown to to contact neurons (sketch of some generic shape), instead of going into some abstract circular hole

2. For imaging side, instead of camera icon, an objective could be shown. Also, here some sketch of typical acquired image could be shown.

3. For drug screening part, the application flow of drug-containing solution might be shown instead of stationary test-tubes

The paper is very good as it is (apart from usual recommendations to double-check for typos and minor language errors)

One could recommend to focus in the title and abstract on reliability and reproducibility, rather then on low-cost. For many labs, especially in USA/Europe, “no-fuss” and “easy-made” is much more important than low-cost.

Also, significant part of the manuscript shows results of experiments carried out with the chamber

So, one might suggest to change the title for “ Simple and efficient system for in vivo electrophysiological and imaging studies in rodents"

Reviewer #2

It is highly desirable to perform intrinsic optical imaging, intra-, and extracellular recordings under stable conditions in vivo. This important and interesting study demonstrates that a novel 3D-printed superfusion chamber allows these long-lasting recordings in spatially restricted neocortical regions of young (P4-7) and adolescent (P20-27) urethane-anesthetized rats. This chamber can be easily fabricated and can be most valuable for future studies on cortical physiology.

I have a few questions and comments the authors should address:

1) Is it possible to perform chronic recordings with this chamber over days?

2) I understand that the statement “the superfusion chamber in vivo does not modify the physiological patterns of activity”. Is this true for upper and lower cortical layers?

3) Can this chamber be modified to fit to the (newborn, young) mouse?

4) Please comment on the wash-in and wash-out times of drugs applied via the chamber?

5) I find the lower panels of Fig. 3E-G and the right panel of Fig. 4B not very helpful.
